# Rifampicin for the treatment of a cardiogenic shock related to mavacamten toxicity: the worse and the best of drug interactions from a case report

**DOI:** 10.1093/ehjcr/ytag368

**Published:** 2026-05-14

**Authors:** Simon Lucas, Benjamin Hennart, Pascal de Groote, Nicolas Lamblin, Helene Ridon, Celine Dupre, Arnaud Lionet, Gilles Lemesle

**Affiliations:** Cardiology Department, Heart and Lung Institute, University Hospital of Lille, CHU Lille, Bd du Pr Jules Lecle rcq, 59000 Lille, France; Toxicology and Genopathy Department, University Hospital of Lille, CHU Lille, Avenue Oscar Lambret, 59000 Lille, France; Université de Lille, rue Paul Duez, 59000 Lille, France; ULR 4483 - IMPECS - IMPact de L’Environnement Chimique sur la Santé Humaine, rue Paul Duez, 59000 Lille, France; Cardiology Department, Heart and Lung Institute, University Hospital of Lille, CHU Lille, Bd du Pr Jules Lecle rcq, 59000 Lille, France; Institut Pasteur of Lille, Inserm U1167, rue du Pr Calmette, 59000 Lille, France; Cardiology Department, Heart and Lung Institute, University Hospital of Lille, CHU Lille, Bd du Pr Jules Lecle rcq, 59000 Lille, France; Université de Lille, rue Paul Duez, 59000 Lille, France; Institut Pasteur of Lille, Inserm U1167, rue du Pr Calmette, 59000 Lille, France; Department of Cardiovascular Explorations, Heart and Lung Institute, University Hospital of Lille, Bd du Pr Jules Leclercq, 59000 Lille, France; Department of Anesthesia and Intensive Care, Heart and Lung Institute, University Hospital of Lille, Bd du Pr Jules Leclercq, 59000 Lille, France; Université de Lille, rue Paul Duez, 59000 Lille, France; Department of Nephrology, University Hospital of Lille, Avenue Oscar Lambret, 59000 Lille, France; UMR9020-U1277-CANTHER-Cancer Heterogeneity Plasticity and Resistance to Therapies, CNRS, Inserm, rue Paul Duez, 59000 Lille, France; Cardiology Department, Heart and Lung Institute, University Hospital of Lille, CHU Lille, Bd du Pr Jules Lecle rcq, 59000 Lille, France; Université de Lille, rue Paul Duez, 59000 Lille, France; Institut Pasteur of Lille, Inserm U1011, rue du Pr Calmette, 59000 Lille, France; French Alliance for Cardiovascular Trials (FACT), Paris, France; Service USIC et Centre Hémodynamique, Institut Cœur Poumon, Bd du pr Jules Leclercq, CHU de Lille, 59037 Lille Cedex, France

**Keywords:** Cardiogenic shock, Hypertrophic cardiomyopathy, Mavacamten, Cytochrome CYP2C19, Drug interactions, Rifampicin, Case report

## Abstract

**Background:**

Mavacamten has been shown to be efficient in the treatment of obstructive hypertrophic cardiomyopathy (HOCM). However, its use may be associated with toxicity and subsequent adverse events. *CYP2C19* genotyping is therefore recommended to optimize dosing and minimize risks in daily practice.

**Case summary:**

We report the case of a 39-year-old woman with a history of sleeve gastrectomy and HOCM. She was initiated on mavacamten 5 mg once daily *(CYP2C19* genotyping showed no mutation) because of persistent significant obstruction and dyspnoea (NYHA class II) under nebivolol. Although initial tolerance was good with a reassuring transthoracic echocardiography at 4 months, she experienced a severe cardiogenic shock related to mavacamten overdose in the context of esomeprazole initiation (proton pump inhibitor that strongly interacts with the cytochrome CYP2C19 and CYP3A4) for an oesophageal pyrosis by her general practitioner 8 months later. Notably, the toxicity could be completely overcome by rifampicin initiation (600 mg twice daily for 3 days), which allowed the normalization of the plasma level of mavacamten within a few days and patient recovery.

**Discussion:**

The present case highlights that drug interactions in patients with HOCM receiving mavacamten are critical and may lead to a high level of toxicity and severe heart failure events, including cardiogenic shocks. Patient and caregiver information about these potential interactions is highly important. This case also emphasizes that the dosage of mavacamten is now available (and should be used in specific situations) and that mavacamten toxicity can rapidly be overcome by early rifampicin administration, a strong hepatic enzyme inducer.

Learning points

- Recognition of drug interactions is critical in patients with hypertrophic cardiomyopathy under mavacamten therapy.
- Mavacamten toxicity may lead to severe cardiogenic shock even at the distance of treatment initiation.
- Dosage of plasma level of mavacamten is advised in specific clinical situations, when overdose may occur.
- CYP2C19 and CYP3A4 induction by rifampicin is an effective treatment for mavacamten overdose.

## Introduction

Obstructive hypertrophic cardiomyopathy (HOCM) is a myocardial disorder characterized by left ventricular (LV) hypertrophy usually resulting from sarcomeric gene mutations.^1,2^ Progressive diastolic dysfunction and fibrosis contribute to heart failure (HF) and ventricular arrhythmias, which may lead to sudden death.

In HOCM, relief of obstruction has been shown to improve both symptoms and prognosis.^1–4^ First-line medical therapy includes β-blockers and non-dihydropyridine calcium channel blockers.^1,2,5^ Disopyramide may be added for persistent obstruction and high gradients.^1,2,6^ When medical therapy fails, septal reduction therapies, surgical myectomy, or alcohol septal ablation remain an effective but invasive options.^1,2,7^

Recently, mavacamten, a first-in-class selective cardiac myosin ATPase inhibitor, directly targeting the molecular hypercontractility of HOCM, has shown efficacy in obstruction relief.^8–10^ By reducing the number of myosin heads interacting with actin, it decreases left ventricular outflow tract (LVOT) gradient, improves diastolic filling, and may reverse adverse LV remodelling. In the EXPLORER-HCM trial, mavacamten significantly improved exercise capacity, NYHA functional class, and LVOT gradient compared to placebo, with sustained benefits observed in long-term extension studies.^11^ Recent meta-analyses have confirmed reduction in resting and provoked gradients (40–55 mmHg decrease in average) and improvement in biomarkers and cardiac structure.^1,2,12^ Current guidelines therefore recommend mavacamten use for patients with symptomatic HOCM who remain limited despite optimal conventional therapy and who might otherwise undergo septal reduction.^1,2^ While long-term mortality and arrhythmic outcomes remain under study, mavacamten represents a major therapeutic advance by addressing the fundamental pathophysiology of sarcomeric hypercontractility and offering a non-invasive alternative for relief of obstruction and symptom burden in HOCM.

Nevertheless, mavacamten use is associated with side effects and risks.^12^ Because it is metabolized primarily and almost exclusively by the cytochrome CYP2C19 (≈75%), the poor-metabolizer genotype leads to markedly increased area under the curve in the blood and prolonged half-life.^12^ Hence, *CYP2C19* genotyping is recommended to optimize dosing and minimize risks in daily practice. Of note, in patients with CYP2C19 deficiency, mavacamten may alternatively be metabolized by the cytochromes CYP3A4 and CYP3A5. Critically, drug interactions should therefore be managed with strong caution, and close echocardiographic monitoring is essential to detect reversible reduction in left ventricular ejection fraction (LVEF).

The present case is a perfect illustration of both the efficacy and safety issues of mavacamten use in HOCM patients.

## Case presentation

**Figure 1:**
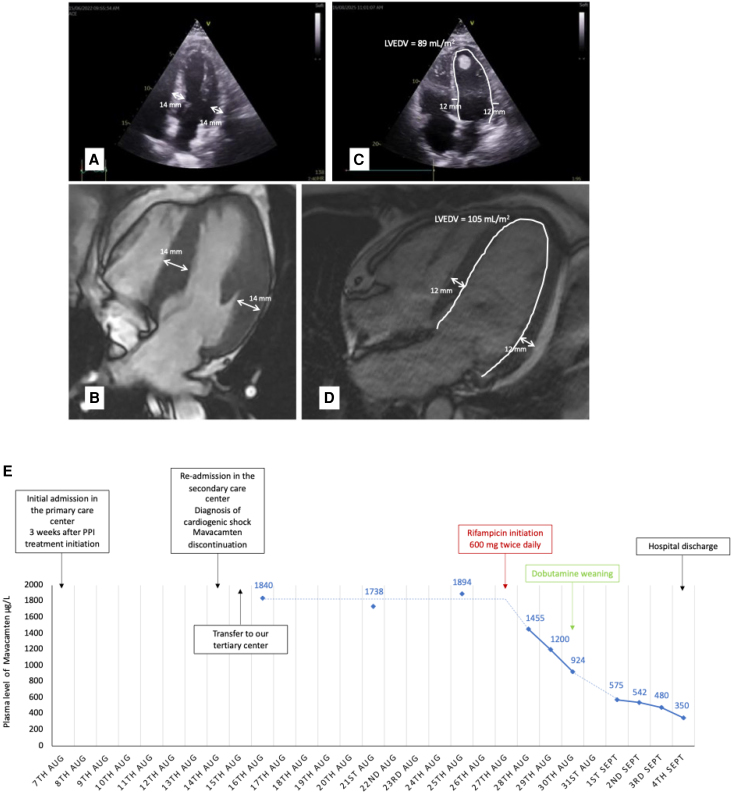
A young woman with cardiogenic shock associated with mavacamten overdose. A: Initial transthoracic echocardiography (TTE) performed in 2022 at the early stage of the HOCM disease and before the initiation of mavacamten [four-chamber view showing a symmetric left ventricle (LV) hypertrophy at 14 mm]. B: Initial cardiac magnetic resonance imaging (MRI) performed in 2021 at the time of the initial diagnosis (four-chamber view showing a symmetric LV hypertrophy at 14 mm). C: New TTE performed in August 2025 at the time of cardiogenic shock (four-chamber view showing a significant and symmetric reduction in LV hypertrophy at 12 mm associated with LV dilation and the presence of an apical LV thrombus). D: New cardiac MRI performed in August 2025 at time of cardiogenic shock (4-chamber view showing a significant and symmetric reduction in LV hypertrophy at 12 mm and a strong alteration of both LV and right ventricle (RV) functions associated with LV dilation. E: Kinetics of plasma levels of mavacamten during the hospitalization for cardiogenic shock, before and after rifampicin initiation (dotted lines show “virtual” evolution of plasma level in the absence of dosage, dosages on August 16th and August 21st were performed a posteriori).

In 2021, a 39-year-old woman was diagnosed with HOCM in the context of a cardiovascular evaluation before a sleeve gastrectomy for the treatment of severe obesity [body mass index (BMI) at 35 kg/m^2^]. She had hypertension (not treated) and a mild obstructive sleep apnoea syndrome that did not require any dedicated treatment. Initial transthoracic echocardiography (TTE) revealed a symmetric and mild LV hypertrophy at 14 mm. The LVEF was 70%, and the maximal peak jet velocity was 2.1 m/s in the LVOT (no obstruction) (*Figure 1A*). Initial cardiac MRI confirmed the diagnosis (*Figure 1B* and Supplementary material online, *Video S1*) and also revealed mild fibrosis of the posterior mitral papillary muscle. She was initially initiated with nebivolol 10 mg once daily and remained clinically stable for several years with no acute event, neither heart failure nor arrhythmia. Of note, no gene mutation (for HOCM) was found. The sleeve gastrectomy was performed with success under nebivolol treatment in 2021.

Four years later, in October 2024, her BMI was 27 kg/m^2^. However, since she experienced worsening in the shortness of breath (NYHA class II), a new TTE was performed, which revealed significant deterioration. Although the LVEF remained normal at 70% with no kinetic abnormalities, LV hypertrophy worsened drastically with a septum thickness of 23 mm and a posterior wall of 20 mm. In addition, a significant systolic anterior motion of the anterior mitral valve leaflet appeared, leading to severe LVOT obstruction with a maximal peak jet velocity at 6.1 m/s. The left atrium was mildly dilated at 45 mL/m^2^.

She was therefore referred to our tertiary centre for further evaluation and to discuss the initiation of mavacamten. As recommended, *CYP2C19* genotyping was performed and revealed a normal CYP2C19 metabolizer phenotype; subsequently, normal hepatic metabolism was expected. Mavacamten was initiated at 5 mg once daily on top of nebivolol on January 27, 2025. Instructions for the risk of drug interactions were given to the patient. A control TTE was performed on May 2025 and showed a slight decrease in LV hypertrophy with a septum at 21 mm and a posterior wall at 18 mm. Importantly, there was no more significant LVOT obstruction with a peak jet velocity at 1.2 m/s and no more systolic anterior motion of the mitral valve. The LVEF remained normal at 60%. Clinically, the patient improved significantly with no dyspnoea at that time.

On 7 August 2025, the patient was admitted to a primary care centre for abdominal pain and vomiting. Initial evaluation concluded to a case of gastroenteritis, although the biology showed a severe increase in hepatic enzymes (10 times the upper limit), and the patient was discharged on the same day. Seven days later, she was re-admitted to a secondary care centre with severe haemodynamic impairment and cardiogenic shock. The biology revealed an increase in the lactate level at 5 mmol/L and in hepatic enzymes that were 15 times above the upper limit. The estimated glomerular filtration rate (eGFR) was still normal at 75 mL/min (Supplementary material online, *Table S1*). TTE revealed a sharp decrease in LVEF at 10% with global severe hypokinesia. The LV was dilated at 89 mL/m^2^ (LV indexed volume), and LV hypertrophy completely disappeared with a septum and a posterior wall thickness of 12 mm. Importantly, an apical thrombus (15 × 19 mm) and a severe right ventricle (RV) dysfunction were also observed (*Figure 1C*). Mavacamten and nebivolol were both immediately discontinued (August 14). Initially, the patient reported no recent modification in her medicines and no error or voluntary increase in drug intake. Dobutamine was initiated, which allowed us to stabilize the patient who was then transferred to our tertiary centre for further evaluation and discuss invasive management (cardiac assistance and heart transplantation).

At admission, the patient was stabilized under dobutamine 10 gamma/kg/min. Initial TTE confirmed severe LV and RV dysfunctions. An invasive right heart catheterization was performed under the same dose of dobutamine on 19 August 2025 and showed a low cardiac index at 1.9 L/min/m^2^ and no congestion or pulmonary hypertension. The right atrium pressure was 4 mmHg (no systemic congestion), the pulmonary pressure was 27/11 mmHg (mean 20 mmHg), and the wedge pressure was 9 mmHg (no pulmonary congestion). Vascular pulmonary resistances were calculated at 3.4 wood units. After an increase in the dobutamine dose to 20 gamma/kg/min during catheterization, the cardiac index improved to 2.6 L/min/m^2^, and the right atrium and wedge pressures decreased to 1 and 5 mmHg, respectively.

The patient remained stable for several days, although not really improving. The eGFR remained normal, and the lactate level returned to normal values within 2 days, but hepatic enzymes slowly decreased to 5 times the upper limit within 9 days (Supplementary material online, *Table S1*). Failure to wean dobutamine occurred twice between admission and 27 August 2025.

Within the first days, aside from stabilizing the patient, we looked for an aetiology that may explain this acute and severe cardiogenic shock since everything was quite normal a few months earlier. First, although an acute coronary event may not be the explanation, a systematic coronary angiography was performed, and it was normal. A new cardiac MRI was also performed on August 25 and confirmed severe LV (LVEF at 10%) and RV (RVEF at 17%) dysfunctions (*Figure 1D* and Supplementary material online, *Video S2*). The LV was dilated, evaluated at 105 mL/m^2^ (LV indexed volume). LV thrombus disappeared under heparin therapy. Septum and posterior wall thicknesses were at 13 mm (LV mass was calculated at 109 g/m^2^). No extended fibrosis or oedema was observed. Acute myocarditis was therefore excluded. Because the patient did not improve enough after a week, no explanation could be found, and although mavacamten was immediately stopped at the time of cardiogenic shock diagnosis, it was decided to dose the residual plasma level of mavacamten on August 25. Noteworthy, the plasma level of mavacamten was still very high after 11 days of cessation and measured at 1894 μg/mL, twice the level of toxicity (therapeutic level between 300–700 μg/mL—toxicity cutoff at 1000 μg/mL). Since then, the cardiogenic shock aetiology appeared to be clearly associated with mavacamten toxicity. A very meticulous discussion with the patient was renewed about any medicine modifications within the last weeks and finally revealed that on 15 July 2025 (3 weeks before the first admission), the patient was initiated by her general practitioner a proton pump inhibitor (esomeprazole 20 mg once daily), which strongly interacts with CYP2C19 (−43%) and CYP3A4 (−28%) cytochromes, for 15 days (last dose July 31) because of oesophageal pyrosis.

After a multidisciplinary discussion including cardiologists, nephrologists, toxicologists, and critical care physicians, several hypotheses were listed in order to improve the patient. Of note, liver functions remained partially impaired. Importantly, close to 98% of mavacamten binds to protein (especially albumin) in the blood, which limits the possibility of removing it from the blood by conventional dialysis. A plasmapheresis is therefore mandatory, but the haemodynamic status of the patient was felt to be too unstable. On August 27, it was therefore decided to start rifampicin 600 mg twice daily for 3 days and check the hemodynamic status and plasma level of mavacamten every day (*Figure 1E*). Within 3 days, the plasma level decreased to 924 μg/mL, the patient improved, and dobutamine could be completely weaned on August 30. A blood sample, which was taken at admission on August 16, was retrieved and revealed that the plasma level was as high as on August 25 at 1840 μg/mL (*Figure 1E*), suggesting the absence of any clearance of the drug, although completely stopped for 9 days.

The patient finally recovered within a few days and was discharged on September 4 without any medicines (nebivolol and mavacamten were not reinitiated). Discharge TTE showed a partial but significant LVEF recovery at 35% and no LVOT obstruction. Other parameters were not significantly modified, and LV dilation remained. The case was declared on the international Vigilyze@ website. Two months after discharge, LVEF was normal, but LV hypertrophy (septum 18 mm, posterior wall 13 mm) and LVOT obstruction (3.94 m/s) both reappeared. Bisoprolol 5 mg once daily was therefore re-initiated.

## Discussion

Although efficient for obstruction relief in HOCM patients, it is well established that mavacamten may lead in some cases to a significant decrease in LVEF and heart failure.^1,2,11,12^ Such events usually occurred within 1–3 months after initiation, therefore requiring early TTE control.^1,2,12^ If it sometimes leads to heart failure events, cardiogenic shock has been very rarely reported, and only four cases have been described on the Vigilyze@ platform and in the literature so far. Of note, one of these cases was recently published and was also related to drug interaction (clarithromycin).^13^ Our case is original in many ways.

First, mavacamten toxicity appeared at a distance (8 months) after initiation and was induced by drug interaction with esomeprazole, which strongly interacts with both cytochromes CYP2C19 and CYP3A4. Not only the patient's but also the caregiver's information is critical to avoid this risk. Of note, the sleeve gastrectomy may have played a key role in our case. It is indeed well documented that drug absorption is not linear (may increase or decrease significantly) in these patients. As an example, Kingma *et al.* reported significant variation of plasma lithium levels in patients with a history of bariatric surgery.^14^ In such patients, it is recommended to repeatedly check for drug plasma levels when treatments with narrow therapeutic targets are initiated. The half-life of mavacamten was reported to be quite long and may vary between 9 and 23 days, depending on several variables, including *CYP2C19* genotype. In our case, no mavacamten clearance occurred after 9 days of discontinuation. Importantly, if mavacamten overdose has initially been induced by esomeprazole interaction, cardiogenic shock, and subsequent severe liver dysfunction may have been the cause of a vicious circle, limiting drug clearance. In addition, it is possible that the drug accumulated within tissues and is then released from the intracellular to the extracellular compartment.

Second, this case highlights that the dosage of mavacamten is now available in daily practice and that in case of overdose suspicion, physicians must not hesitate to ask for a plasma level evaluation.

Third, because of severe haemodynamic impairment and a life-threatening situation, it was deemed mandatory to try to withdraw mavacamten from the blood in order to avoid the need for cardiac assistance and/or heart transplantation. Importantly, because mavacamten binds to protein (especially albumin) in the blood, it is impossible to clear it using conventional dialysis. Plasmapheresis is, in fact, the only way to clear mavacamten from the blood, but the haemodynamic status of the patient was felt to be too unstable. Nephrologists came up with the idea to use rifampicin, a strong non-selective enzyme inducer, as it is used in tacrolimus overdose in patients with renal transplantation, in order to help with mavacamten clearance.^15–17^ The decision was therefore made to try this strategy for 3 days, and it was shown to be very efficient, as illustrated by the present case. Indeed, the plasma level of mavacamten rapidly decreased by 50% within 3 days. It is, however, important to note that when initiating rifampicin, it is also very important to check if other drug interactions may occur in a deleterious fashion.

Finally, based on the presence of tachycardia, low blood pressure, low PT level, hepatic cytolysis predominantly affecting TGO at initial admission, and the presence of clinical signs of shock, very poor LV and RV functions, low cardiac output, and high lactate level at the second admission, we feel that hepatic cytolysis was clearly related to cardiogenic shock. In addition, although hepatic cytolysis (the only biological abnormality) has been described in 2%–3% of cases, no clinical drug-induced hepatitis has been described so far, making it very unlikely that this occurred in our case.

## Conclusion

The present case highlights that drug interactions in patients with HOCM receiving mavacamten are critical and may lead to high levels of toxicity and severe heart failure, including cardiogenic shock. Recognition of this risk is of main importance in daily practice. Importantly, this case also emphasizes for physicians that this toxicity can rapidly be overcome in daily practice by early rifampicin administration, a strong hepatic enzyme inducer. To our knowledge, this is the first time that this effect is described.

## Supplementary Material

ytag368_Supplementary_Data

## Data Availability

The data underlying this article are available in the manuscript.

## Lead author biography

Professor of Cardiology at the Heart and Lung Institute, University Hospital of Lille, France. He is interventional cardiologist and head of the intensive cardiac care unit. Involved in research, chair of the group for research development at the French Society of Cardiology (GRRC: Goupe de Réflexion sur la Recherche Cardiovasculaire) and co-chair of the FACT group (French Alliance for Cardiovascular Trials, F-CRIN network). Member of the European Society of Cardiology (ESC) and of the European Association of Percutaneous Coronary Intervention (EAPCI). Member of the editorial board of the Archives of Cardiovascular Diseases and the European Journal of Preventive Cardiology journals.

## Notes

**Consent:** The patient gave her consent for using her personal data in the present publication. The European Heart Journal—Case Reports Patient Consent Form was used and is available upon request.
