# Supplementary material for: Rifampicin for the treatment of a cardiogenic shock related to mavacamten toxicity: the worse and the best of drug interactions from a case report
Source: Eur Heart J Case Rep. 2026 May 14;10(5):ytag368. doi: 10.1093/ehjcr/ytag368 (PMC13270335; doi:10.1093/ehjcr/ytag368)
Supplement: ytag368_Supplementary_Data [file ytag368_Supplementary_Data.zip › Supplemental Table 1.docx]

**Supplemental Table 1.** Vital signs and biology overtime.

| **Parameters** | **Aug. 7^th^** | **Aug. 8^th^** | **Aug. 9^th^** | **Aug. 10^th^** | **Aug. 11^th^** | **Aug. 12^th^** | **Aug. 13^th^** | **Aug. 14^th^** | **Aug. 15^th^** | **Aug. 16^th^** | **Aug. 17^th^** | **Aug. 18^th^** | **Aug. 19^th^** | **Aug. 20^th^** | **Aug. 21^st^** | **Aug. 22^nd^** | **Aug. 23^rd^** | **Aug. 24^th^** | **Aug. 25^th^** | **Aug. 26^th^** | **Aug. 27^th^** | **Aug. 28^th^** | **Aug. 29^th^** | **Aug. 30^th^** | **Aug 31st** | **Sep. 1^st^** | **Sep. 2^nd^** | **Sep. 3^rd^** | **Sep. 4^th^** |
| --- | --- | --- | --- | --- | --- | --- | --- | --- | --- | --- | --- | --- | --- | --- | --- | --- | --- | --- | --- | --- | --- | --- | --- | --- | --- | --- | --- | --- | --- |
| **Vital signs** |  |  |  |  |  |  |  |  |  |  |  |  |  |  |  |  |  |  |  |  |  |  |  |  |  |  |  |  |  |
| Heart rate, bpm | 128 |  |  |  |  |  |  | 137 | 120 | 116 | 121 | 137 | 126 | 125 | 127 | 126 | 117 | 113 | 121 | 127 | 126 | 123 | 112 | 106 | 103 | 99 | 97 | 93 | 89 |
| Bloop pressure, mmHg | 99/76 |  |  |  |  |  |  | 88/67 | 100/65 | 99/65 | 98/63 | 85/57 | 101/79 | 102/75 | 83/53 | 97/73 | 108/78 | 102/68 | 105/70 | 117/74 | 97/72 | 105/72 | 100/73 | 102/80 | 107/74 | 111/71 | 116/69 | 117/66 | 115/65 |
| SaO_2_, % | 97 |  |  |  |  |  |  | 98 | 95 | 97 | 98 | 96 | 97 | 97 | 95 | 97 | 98 | 98 | 97 | 98 | 97 | 98 | 98 | 99 | 99 | 98 | 99 | 98 | 99 |
| Temperature, °C | 37.1 |  |  |  |  |  |  | 36.9 | 37.0 | 37.3 | 37.0 | 37.1 | 36.3 | 36.8 | 36.6 | 36.8 | 37.1 | 37.3 | 37.2 | 37.4 | 37.1 | 37.1 | 37.0 | 36.9 | 37.1 | 37.0 | 37.3 | 37.1 | 37.0 |
| Clinical signs of shock | Not reported |  |  |  |  |  |  | Yes | No | No | No | Yes | No | No | Yes | No | No | No | No | No | No | No | No | No | No | No | No | No | No |
|  |  |  |  |  |  |  |  |  |  |  |  |  |  |  |  |  |  |  |  |  |  |  |  |  |  |  |  |  |  |
| **Biology** |  |  |  |  |  |  |  |  |  |  |  |  |  |  |  |  |  |  |  |  |  |  |  |  |  |  |  |  |  |
| Hemoglobin, g/dL | 13.9 |  |  |  |  |  |  | 13.9 | 13.4 | 13.7 | 13.3 | - | - | - | 13.5 | 13.6 | 13.2 | - | - | - | 12.2 | 11.5 | - | 11.6 | - | 11.8 | - | 12.1 | 12.0 |
| Creatinine, mg/L | 10.0 |  |  |  |  |  |  | 10.0 | 8.0 | 7.0 | 7.0 | 7.0 | 7.0 | 6.0 | 5.0 | 5.0 | 5.0 | 5.0 | 6.0 | 6.0 | 5.0 | 5.0 | 4.0 | 4.0 | 5.0 | 6.0 | 6.0 | 6.0 | 7.0 |
| TGO, UI/L | 226 |  |  |  |  |  |  | 2663 | 2039 | 1450 | 931 | 725 | 435 | 294 | 253 | 208 | 143 | 108 | 88 | 76 | 76 | 75 | 54 | 40 | 33 | 28 | 30 | 25 | 30 |
| TGP, UI/L | 410 |  |  |  |  |  |  | 308 | 272 | 355 | 151 | 87 | 55 | 44 | 97 | 99 | 66 | 46 | 43 | 39 | 43 | 40 | 27 | 22 | 18 | 24 | 30 | 31 | 27 |
| $\gamma$GT, UI/L | 275 |  |  |  |  |  |  | 145 | 113 | 271 | 239 | 251 | 203 | 188 | 188 | 211 | 223 | 230 | 260 | 274 | 294 | 288 | 263 | 228 | 232 | 239 | 210 | 191 | 173 |
| Total bilirubin, mg/L | 9 |  |  |  |  |  |  | 26 | 24 | 28 | 31 | 39 | 36 | 28 | 26 | 25 | 22 | 17 | 15 | 14 | 12 | 20 | 24 | 15 | 9 | 6 | 7 | 7 | 5 |
| Alkaline phosphatase, UI/L | 84 |  |  |  |  |  |  | - | - | 106 |  |  |  |  |  |  |  |  |  |  |  |  |  |  |  |  |  |  |  |
| Lipase, UI/L | 35 |  |  |  |  |  |  | - | - | 121 |  |  |  |  |  |  |  |  |  |  |  |  |  |  |  |  |  |  |  |
| PT, % | 51 |  |  |  |  |  |  | 25 | 31 | 43 | 64 | 67 | 75 | 75 | 78 | 73 | 71 | 72 | 76 | 76 | - | - | 82 | - | - | - | - | 85 | 89 |
| Factor V, % | 57 |  |  |  |  |  |  | 28 | 31 | 36 | 58 | 78 | 90 | 101 | 109 | 119 | 132 | 122 | 126 | 123 | - | - | 124 | - | - | - | - | 142 | 129 |
| Lactates, mmol/L | - |  |  |  |  |  |  | 5.6 | 3.0 | 2.2 | 2.0 | 1.5 | 1.0 | 1.5 | 1.3 | 1.7 | 1.4 | 0.9 | 0.9 | 1.0 | 0.9 | 1.0 | 0.8 | 0.9 | - | - | - | - | - |
| ScvO_2_, % | - |  |  |  |  |  |  | 57 | 51 | 50 | 58 | 66 | 69 | 63 | 44 | 58 | 61 | 63 | 61 | 60 | 65 | 75 | 82 | 79 | - | - | - | - | - |
| NT-pro-BNP, pg/mL | 2692 |  |  |  |  |  |  | 1964 | 1525 | 799 | 561 | - | - | - | 1253 | - | 1141 | - | 1190 | 1195 | 1143 | 853 | 955 | 990 | 823 | 819 | 884 | 826 | - |
| CRP, mg/L | 107 |  |  |  |  |  |  | 82 | 63 | 59 | - | - | - | - | - | - | 90 | - | - | - | 57 | - | - | 35 | - | 17 | - | 12 | 10 |

PT = prothrombin time, Sa0_2_ = arterial saturation, ScvO_2_ = central venous oxygen saturation

Grey boxes display the period when the patient was not hospitalized.
